# Early Experience with Intravitreal Bevacizumab Combined with Laser Treatment for Retinopathy of Prematurity

**DOI:** 10.4103/0974-9233.80716

**Published:** 2011

**Authors:** Seemant Raizada, Jamal Al Kandari, Ahmad Al Foudari

**Affiliations:** Al Bahar Eye Centre, Ibn Sina Hospital, Kuwait

Sir,

We read with interest the article by Ahmed and colleagues^1^ We are members of the vitreoretina team of surgeons of the Retinopathy of Prematurity (ROP) program in Kuwait.^2^ We screen over 100 premature neonates weekly and deal with their treatment and follow-up. Bevacizumab (commonly referred to as *Avastin*™), Genentech Inc, San Francisco, CA, USA reportedly has potential off-label use for treating conditions related to Vascular Endothelial Growth Factor (VEGF) excesses, eg, Age-related macular degeneration, retinal vascular occlusions, diabetic macular edema, etc. Ahmed and colleagues^1^ report their experience with intravitreal Bevacizumab for ROP.

We would like to point out the following regarding this fine study:

1. This is a case series on simultaneous use of Bevacizumab and laser therapy in the same sitting for ROP in 15 eyes of 8 infants. The inclusion criteria are not clear. The authors state *“All eyes had stage 3 to stage 4 ROP and were at a high risk of permanent vision loss and decreased likelihood of improvement with conventional laser therapy alone.”* How did the authors determine which child is not likely to respond to conventional laser therapy? As we understand from the article, there was, neither a prior trial of laser nor a reasonable evaluation period before Bevacizumab was injected. Currently, standard-of-care for ROP is timely laser therapy, with the majority of cases responding adequately, even for aggressive ROP. We believe that a trial of laser therapy should be performed for ROP before trying a new treatment modality. There is a relative paucity of published studies evaluating the effect of Bevacizumab (anti-VEGF agent primarily indicated for colorectal carcinoma) on the pediatric retina. Additionally, the long-term systemic effects of this drug on neonates are not known. However, Ahmed and colleagues^1^ do state that Bevacizumab can cause delay in normal physiological neovascularization. They used 0.625 mg Bevacizumab for intravitreal injections. Was there any scientific reason to reduce the dose to half or was this reduction due to the fact that neonates were undergoing treatment?
2. We know through experience and peer reviewed papers that intravitreal bevacizumab temporarily decreases leakage from diabetic neovascular lesions, but this treatment may be associated with tractional retinal detachment (TRD).^3^ Even in ROP, one investigator reported an increase in TRD after Bevacizumab injection.^4^ Hence, we believe that serious consideration of the risk of TRD is warranted prior to considering Bevacizumab, especially in stage IV cases included in the study by Ahmed and colleagues^1^ (5 cases).
3. The procedure of intravitreal injection of Bevacizumab should have been well documented in the Material and Methods section, as it is a common practice for peer review studies for others to repeat the study. The authors do not document how they delivered the intravitreal injection and at what distance from the limbus. Intravitreal injection in neonates is complicated due to the unique surgical anatomy of the infant eye. The pars plana first develops during the second trimester of gestation. A rapid growth phase occurs between 26 and 35 weeks post conception. In addition to its small size, the neonatal eye differs from the adult eye with respect to the spatial relationship of intraocular structures. In the neonatal eye, the pars plana region is not fully developed and the anterior retina lies just behind the pars plicata. Hence, utmost care is needed for intravitreal injections because it may cause inadvertent lens touch, traction on the vitreous base, and retinal damage. In the study by Ahmed and colleagues^1^, intravitreal injections were performed under general anesthesia. General anesthestic for neonates, especially premature infants, can be difficult and may lead to grave morbidity and potential mortality. Hence, the risk-benefit ratio should be carefully weighed before considering intravitreal injection for ROP under general anesthesia.
4. It is common knowledge that Bevacizumab decreases neovascular proliferation. However, we do not agree with the conclusion given by Ahmed and colleagues^1^: *“These observations strongly suggest that Bevacizumab effectively reduces vascular activity in ROP.”* The design of their study does not support this conclusion. For example, there was no sham group, nor was there a control group for comparison. As both laser and Bevacizumab were delivered virtually together, the reduced vascularity cannot be solely attributed to Bevacizumab. A number of studies in peer review literature have reported that laser therapy can have the same effect.^5^
5. We commend Ahmed and colleagues in their attempt to differ from the conventional wisdom, but, at the same time, would caution them and the readers of their study that there is scant evidence to justify the routine use of Bevacizumab for ROP. Perhaps, multicenter trials and greater use may provide adequate knowledge regarding the intravitreal Bevacizumab for ROP. However, until there is a greater preponderance of evidence on this topic, it is wise to refrain from using Bevacizumab for ROP. If one must resort to this mode of treatment, we believe it should be reserved only for cases that are refractory to conventional laser therapy or where laser cannot be performed due to extremely hazy media and poor visibility that precludes the use of the laser.

## References

[1] Ahmed AE, Channa R, Durrani J, Ali A, Ahmad K (2010). Early experience with intravitreal bevacizumab combined with laser treatment for retinopathy of prematurity. Middle East Afr J Ophthalmol.

[2] Wani VB, Kumar N, Sabti K, Raizada S, Rashwan N, Shukkur MM (2010). Results of screening for retinopathy of prematurity in a large nursery in Kuwait: Incidence and risk factors. Indian J Ophthalmol.

[3] Nicholson BP, Schachat AP (2010). A review of clinical trials of anti-VEGF agents for diabetic retinopathy. Graefes Arch Clin Exp Ophthalmol.

[4] Kusaka S, Shima C, Wada K, Arahori H, Shimojyo H, Sato T (2008). Efficacy of intravitreal injection of bevacizumab for severe retinopathy of prematurity: A pilot study. Br J Ophthalmol.

[5] (2003). Early Treatment For Retinopathy Of Prematurity Cooperative Group. Revised indications for the treatment of retinopathy of prematurity: Results of the early treatment for retinopathy of prematurity randomized trial. Arch Ophthalmol.

